# Healthcare Professionals’ Perspectives on Age-Friendly Primary Care: Technological and Cultural Challenges

**DOI:** 10.1093/geroni/igaf122.3164

**Published:** 2025-12-31

**Authors:** Lia Ring, Paule-Sarah Fraiman, Yosefa Birati, Reem Nashef-Hamuda, Shoshi Keisari, Anna Zisberg

**Affiliations:** Department of Social Work, Ruppin Academic Center, Emek Hefer, Israel; University of Haifa, Haifa, HaZafon, Israel; The Cheryl Spencer Department of Nursing Faculty of Social Welfare and Health Science, Hanaton, HaZafon, Israel; University of Haifa, Tel Aviv University, Haifa, HaZafon, Israel; University of Haifa, Haifa, HaZafon, Israel; University of Haifa, Haifa, HaZafon, Israel

## Abstract

Older adults face unique healthcare challenges, necessitating age-friendly primary care approaches to better meet their needs, particularly in highly multi-cultural societies like Israel where language and cultural factors may impact healthcare needs and accessibility. Despite practical efforts to improve primary healthcare for older adults the extent to which primary care clinics meet their unique needs remains unclear. This qualitative study explores how healthcare professionals perceive and implement age-friendly adaptations in primary care, focusing on technological and cultural considerations. Semi-structured interviews were conducted in Israel with 21 Jewish and Arab primary healthcare professionals, aged 31 to 69, examining perspectives on clinic adaptations to older patients’ needs. Thematic analysis revealed three main themes regarding the perception of professionals: 1) Age as a Core Identity; 2) Technology, Infrastructure and Administrative Challenges in Primary Care for Older Adults; and 3) Cultural and Linguistic Issues in Older Adult Primary Care. Each category was further defined into specific themes. Examples from each category include: “Challenges and Special Needs Navigating the Healthcare System and Ensuring Continuity of Care”; “The Shift to Digital and Telephone Interfaces for Healthcare Services and Administration”; and “Differences Between Communities”. Although these themes have been discussed, to our knowledge, they haven’t been examined together. The findings provide insights into adaptations to improve primary care for older adults, including tailoring technologies and enhancing communication. This study underscores the importance of language, cultural awareness, and healthcare providers’ age-related perceptions. By integrating these dimensions, this research expands knowledge and offers new perspectives on age-friendly healthcare practices.

